# Burden of bacterial meningitis in India: Preliminary data from a hospital based sentinel surveillance network

**DOI:** 10.1371/journal.pone.0197198

**Published:** 2018-05-16

**Authors:** Yuvaraj Jayaraman, Balaji Veeraraghavan, Girish Kumar Chethrapilly Purushothaman, Bharathy Sukumar, Boopathi Kangusamy, Ambujam Nair Kapoor, Nivedita Gupta, Sanjay Madhav Mehendale, Hospital Based Sentinel Surveillance of Bacterial Meningitis (HBSSBM) Network Team

**Affiliations:** 1 ICMR-National Institute of Epidemiology, Chennai, Tamil Nadu, India; 2 Christian Medical College, Vellore, Tamil Nadu, India; 3 Indian Council of Medical Research, New Delhi, India; Wadsworth Center, UNITED STATES

## Abstract

**Background:**

Worldwide, acute bacterial meningitis is a major cause of high morbidity and mortality among under five children, particularly in settings where vaccination for *H*. *influenzae* type *b*, *S*. *pneumoniae* and *N*. *meningitidis* is yet to be introduced in the national immunization programs. Estimation of disease burden of bacterial meningitis associated with these pathogens can guide the policy makers to consider inclusion of these newer vaccines in the immunization programs. A network of hospital based sentinel surveillance was established to generate baseline data on the burden of bacterial meningitis among children aged less than 5 years in India and to provide a platform for impact assessment following introduction of the Pentavalent and Pneumococcal Conjugate Vaccines (PCV).

**Methods:**

During surveillance carried out in select hospitals across India in 2012–2013, information regarding demographics, immunization history, clinical history, treatment details and laboratory investigations *viz*. CSF biochemistry, culture, latex agglutination and PCR was collected from children aged 1 to 59 months admitted with suspected bacterial meningitis.

**Results:**

A total of 3104 suspected meningitis cases were enrolled from 19,670 children admitted with fever at the surveillance hospitals. Of these, 257 cases were confirmed as cases of meningitis. They were due to *S*. *pneumoniae* (82.9%), *H*. *influenzae* type b (14.4%) and *N*. *meningitidis* (2.7%). Highest prevalence (55.3%) was observed among children 1 to 11 months. Antimicrobial susceptibility testing revealed considerable resistance among *S*. *pneumoniae* isolates against commonly used antibiotics such as cotrimoxazole, erythromycin, penicillin, and cefotaxime. More commonly prevalent serotypes of *S*. *pneumoniae* in circulation included 6B, 14, 6A and 19F. More than 90% of serotypes identified were covered by Pneumococcal Conjugate Vaccine 13.

**Conclusions:**

We observed that *S*. *pneumoniae* was the commonest cause of bacterial meningitis in hospitalized children under five years of age in India. Continued surveillance is expected to provide valuable information and trends in future, to take an informed decision on introduction of pneumococcal vaccination in Universal Immunization Programme in India and will also eventually help in post-vaccination impact evaluation.

## Introduction

Globally, meningitis is a significant cause of morbidity and mortality in the pediatric population accounting for about 180,000 deaths annually [1]. Prior to introduction of vaccines, *Haemophilus influenzae* type *b* (Hib), *Streptococcus pneumoniae*, and *Neisseria meningitidis* were reported to be the commonest causes for bacterial meningitis with their relative contributions varying by time, location and age group in different parts of the world [1, 2]. The burden of bacterial meningitis due to *Hib* and *S*. *pneumoniae* decreased significantly following the introduction of the respective vaccines in high income nations. However, bacterial meningitis continues to be a cause for concern in low and middle income countries either due to low level of vaccine coverage or non- availability of these vaccines in their national immunization programs [1, 3, 4]. Population based studies from South Asian countries and retrospective hospital based studies from these regions have reported that 12.8% and 28% of the confirmed cases of invasive bacterial disease were due to *S*. *pneumoniae*, respectively [5].

Studies from India have attributed pneumonia and meningitis as the leading causes of deaths among children below five years of age accounting together to nearly 22.0% deaths [6]. Hospital based data reports that nearly 40–50% of meningitis and 25–30% of pneumonia cases result from *Hib* infection [7]. In India, multisite studies such as ‘Invasive Bacterial Infection Surveillance’ (IBIS), Alliance for Surveillance of Invasive Pneumococci’ (ASIP), Asian Network for Surveillance for Resistant Pathogens’, Pan Asia Epidemiologic Surveillance Network, Asian Strategy for Pneumococcal Disease Prevention’and few other single site studies have generated data on sero-epidemiology and drug resistance to pneumococcal infections [8]. However, these historical data do not reflect the current trends of pneumococcal meningitis and the current serotypes in circulation. Also, older data have a very limited value in post PCV vaccine impact evaluation in India [8].

From 2012 Government of India introduced Pentavalent Vaccine (DPT+Hep B + Hib) in the Universal Immunization Programme (UIP), across the country in a phased manner and the entire country was covered by 2017 [9]. Baseline data on the prevalence of *H*. *influenzae* type *b* and *S*. *pneumoniae* associated bacterial meningitis and pneumonias by circulating serotypes both in the pre and post vaccine introduction period is vital to assess the impact of the Pentavalent and PCV vaccine introductions in the UIP. Also, such data will be useful for immunization program managers and policy makers to take a decision on revision of the strategy for conjugation of vaccines if any major serotype replacement(s) occur in the post–introduction scenario.

The latest phase of hospital based sentinel surveillance for bacterial meningitis (HBSSBM) was initiated in March 2012 by Indian Council of Medical Research with financial support from the Ministry of Health and Family Welfare (MOHFW), Government of India with the objectives of establishing a surveillance network to generate baseline data on bacterial meningitis at selected sentinel sites, and estimating the prevalence and distribution of bacterial meningitis caused by *H*. *influenzae type b*, *S*.*pneumoniae* and *N*. *meningitidis*. Herein, we describe the framework of the Hospital Based Sentinel Surveillance for Bacterial Meningitis (HBSSBM) network in India and findings from the surveillance activity carried out during 2012–2013.

## Methods

**Fig 1:**
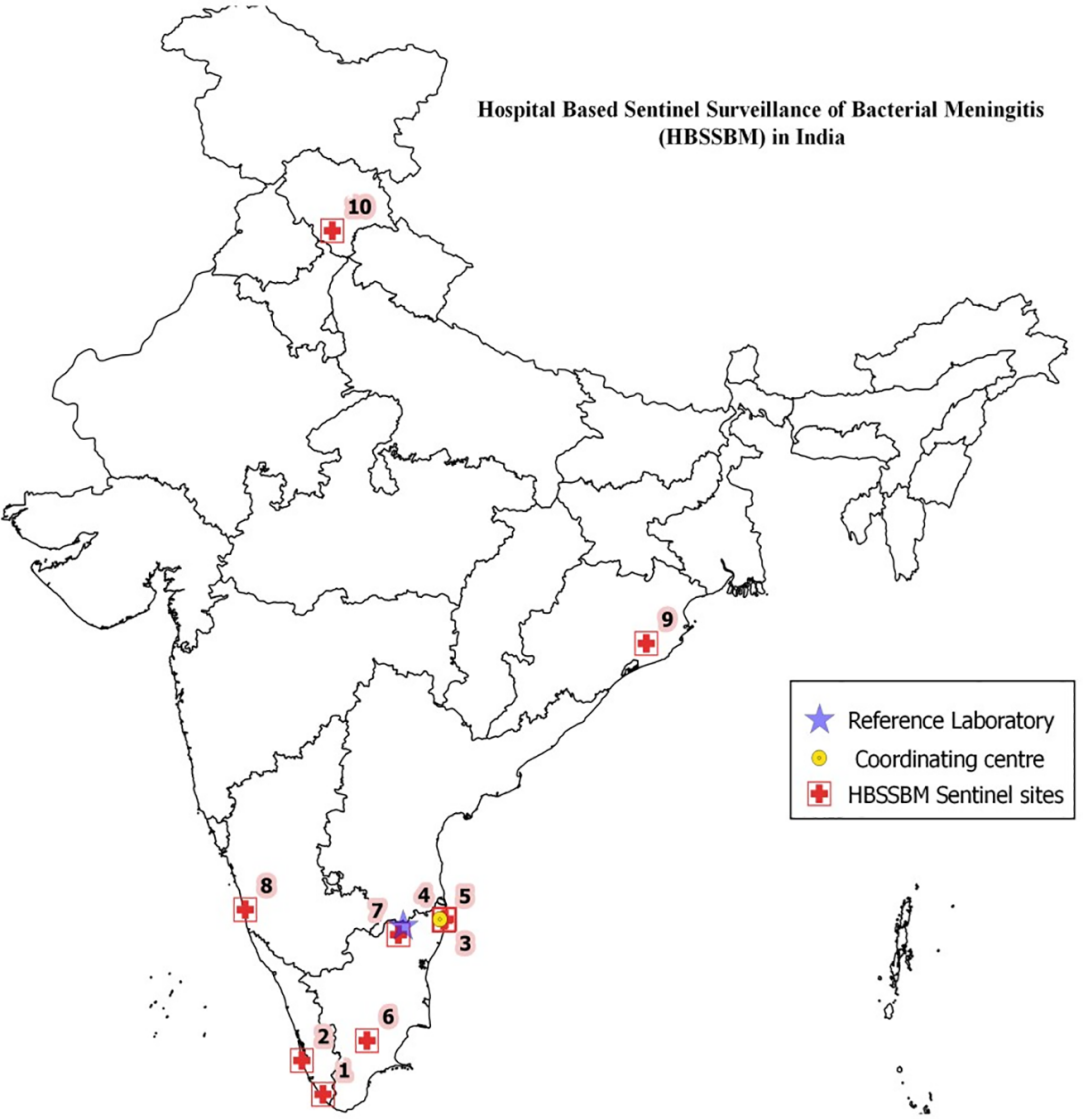
Hospital based sentinel surveillance of bacterial meningitis (HBSSBM) network. 1- Government Medical College, Trivandrum; 2-Government TD Medical College, Allepey; 3- Institute of Child Health, Chennai; 4- Stanley Medical College, Chennai; 5- Kilpauk Medical College, Chennai; 6- Madurai Medical College, Madurai; 7- Christian Medical College, Vellore; 8- Kasturba Medical College and Hospital, Manipal; 9- Regional Medical Research Center, Bhubaneswar; 10- Indira Gandhi Institute of Medical Sciences, Shimla.

HBSSBM was initiated in the year 2012, initially at 10 sentinel sites (Fig 1) in states where Pentavalent Vaccine was first introduced as part of the Universal Immunization Programme. ICMR-National Institute of Epidemiology (NIE), Chennai functioned as the coordinating centre for HBSSBM and rolled out and administered the surveillance activity. Christian Medical College, Vellore served as the Reference Laboratory for the network and administered external quality assurance (EQA) programme for the participating sentinel sites.

### Establishing the surveillance network

The funding of each sentinel site was released after completion of procedural formalities such as approvals from the Institutional Ethics Committees and signing of Memorandum of Understanding between NIE and each of the participating institutions.

### Ethics statement

The present study was carried out in accordance with the Declaration of Helsinki. Specific approvals from the following ethics committees viz. Ethics Committee at ICMR- National Institute of Epidemiology, Chennai; Human Ethics Committee at Government Medical College, Thiruvananthapuram, Kerala; Institutional Ethics Committee at Government TD Medical College, Allepey, Kerala; Institutional Ethics Committee at Institute of Child Health, Chennai, Tamil Nadu; Institutional Ethics Committee at Stanley Medical College, Chennai, Tamil Nadu; Institutional Ethics Committee at Kilpauk Medical College, Chennai, Tamil Nadu; Ethics Committee at Madurai Medical College, Madurai, Tamil Nadu; Institutional Ethics Committee at Kasturba Medical College& Hospital, Manipal, Karnataka; Human Institutional Ethics Committee at ICMR- Regional Medical Research Centre, Bhubaneswar Odisha, Institutional Ethics Committee at Maulana Azad Medical College, New Delhi for study at Chacha Nehru Bal Chikitsalaya Hospital, New Delhi; Ethics Committee at Indira Gandhi Institute of Medical Sciences, Shimla; Institutional Review Board at Christian Medical College and Hospital, Vellore, Tamil Nadu,were obtained for conducting this study at the respective hospitals.Written informed consent was obtained from parents or guardians of children prior to their enrollment in the study.

### The clinical component

#### Study population

Children aged between 1 month and 59 months admitted to the selected sentinel site hospitals with complaints of fever and clinical suspicion of meningitis were eligible for enrollment.

#### Case definitions

Clinically Suspected Meningitis: A child presenting with fever (either by history based on parents’/guardian’s recall or based on clinical findings of body temperature) for a duration of less than seven days along with one of the following signs; neck stiffness, bulging fontanelle, altered or reduced level of consciousness, prostration or lethargy, convulsions without documented seizure disorder.

Clinically Probable Meningitis: A child presenting with suspected meningitis and CSF examination showing turbid appearance, leukocytosis >100 cells/mm^3^ or leukocytosis 10–100 cells/ mm^3^ with either decreased glucose (<40 mg/dl) or elevated protein (>100 mg/dl) levels.

Confirmed meningitis: A case of suspected meningitis with laboratory confirmation for *S*. *pneumoniae*, *H*. *influenzae* type *b* and *N*. *meningitidis* by either CSF culture or latex agglutination or by PCR and/or positive blood culture.

#### Patient enrolment

**Fig 2:**
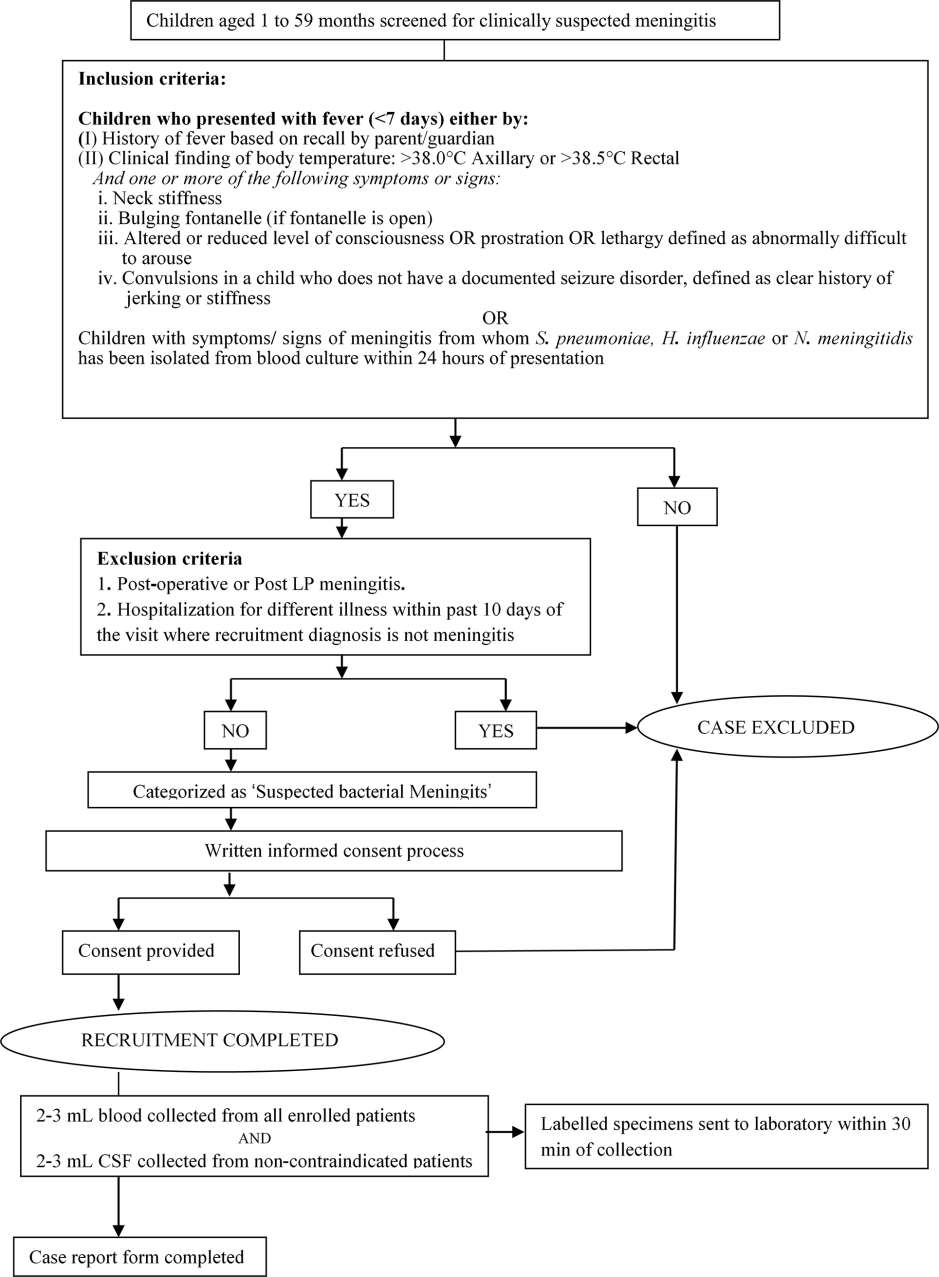
Schematic overview of the case recruitment process in bacterial meningitis surveillance.

Schematic overview of the case recruitment is presented in Fig 2. All children admitted in the in-patient facilities of the participating hospitals with suspected meningitis were enrolled after obtaining written informed consents from their parents or guardians. From each enrolled child, demographic data, pre-admission clinical history, vaccination history, treatment details and outcome details were collected on standardized case report forms.

### Laboratory methods

Under strict aseptic conditions 2–3 mL of venous blood and 2 mL of CSF by spinal tap from the lumbar region were collected. Blood samples were inoculated aseptically at the bed side in blood culture bottles (conventional or automated) and transferred immediately to microbiology laboratory and incubated in a conventional incubator or loaded in automated blood culture system as was applicable. Positive blood cultures were further processed for microbiological confirmation of *H*. *influenzae type b*, *S*. *pneumoniae* and *N*. *meningitidis* following WHO established protocols [10]. Three aliquots of CSF were made, one for microbiological workup (microscopy and culture) and performing latex agglutination test (BD, Directigen^TM^ Meningitis combo test kit, US); second for biochemistry and cytology and third aliquot was stored at-80°C until transportation to reference laboratory for reconfirmation and serotyping. Serotyping for *S*. *pneumoniae* isolates was performed by Quellung reaction using antisera obtained from Staten’s Serum Institute (Copenhagen, Denmark).

For all *S*. *pneumoniae* isolates antimicrobial susceptibility testing (AST) was done by Vitek system (Biomerieux, France) for the following antibiotics: penicillin, cotrimoxazole, erythromycin and cefotaxime as per established WHO protocols and results were interpreted as per the Clinical Laboratory Standards Institute (CLSI) guidelines [10, 11].

### Training

Hands-on induction and annual re-trainings covering clinical, laboratory and data aspects were conducted for all project personnel.

### Quality assurance

External quality assurance was conducted by the reference laboratory, which sent four proficiency panels (each comprising four test strains) to each sentinel site. The EQA results were analyzed and feedback on performance was given to the individual sentinel sites. Periodic monitoring and evaluation visits to each sentinel site were made to review enrolment and other study related protocols.

### Data management

Data entry was performed using Epi-Info (CDC) at each participating site and data was transferred electronically to the coordinating center on a monthly basis. Hard copies of the CRFs were received at the coordinating center for second data entry, data validation and analysis.

### Statistical analysis

Descriptive statistical analysis was performed using SPSS (Version 22.0). Data were presented as counts or percentages.

## Results

**Fig 3:**
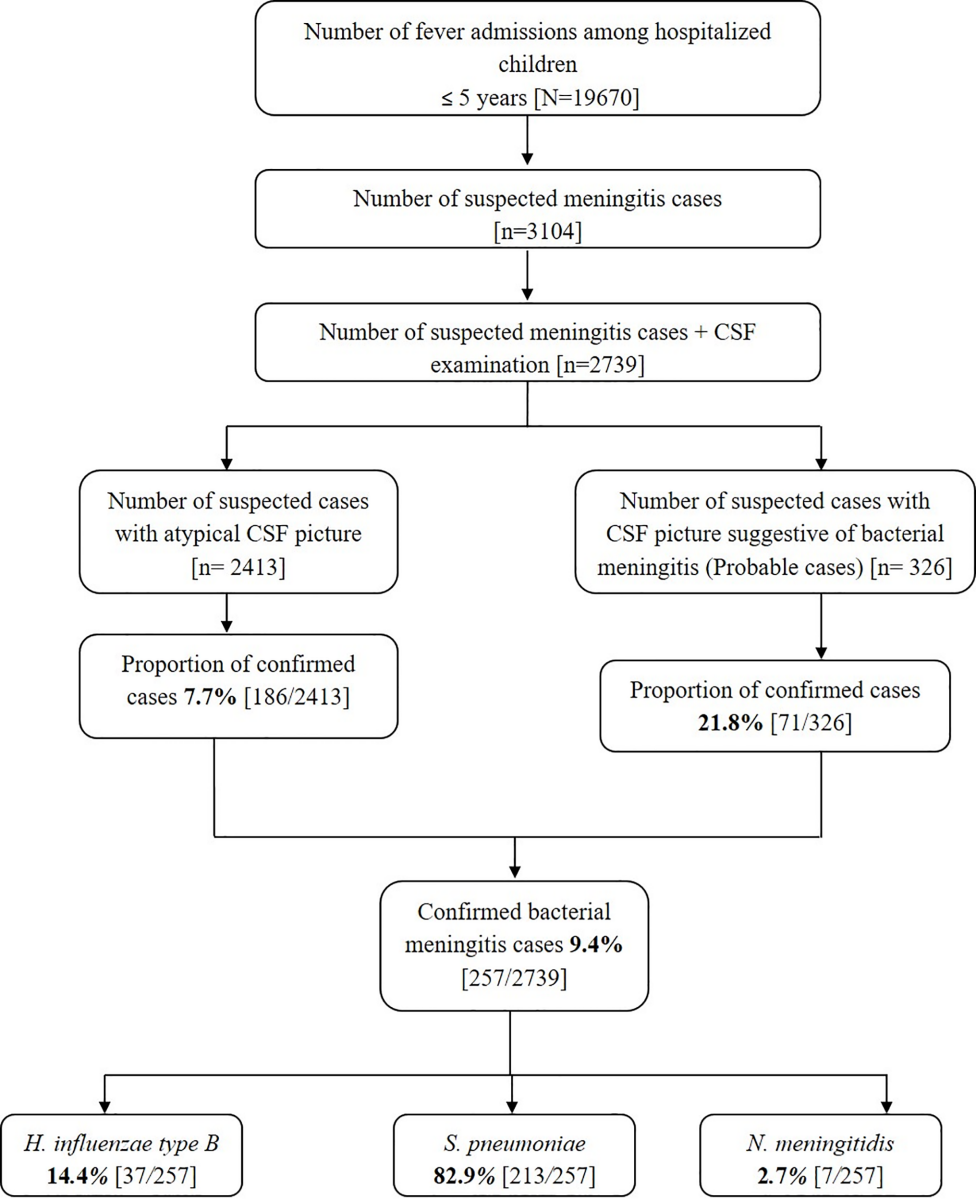
Summary of meningitis case recruitment and diagnostic testing performed during March 2012—February 2013.

A total of 3104 of the 19670 children less than 5 years of age admitted with fever in the 10 sentinel hospitals of the surveillance network in 12 months satisfied the case definition of “suspected bacterial meningitis” (Fig 3).

CSF was available for analysis in 2739 cases of suspected bacterial meningitis. Out of these, 326 (11.9%) cases were labeled as probable meningitis whereas CSF findings were atypical in the remaining 2413 cases. On further laboratory analysis, 21.8% (71/326) of probable cases and 7.7% (186/2413) of atypical suspected cases were confirmed as cases of bacterial meningitis.

In all, 82.9% (213/257) of confirmed cases were positive for *S*. *pneumoniae*, 14.4% (37/257) for *H*. *influenzae* type b and remaining 2.7% (7/257) cases were due to *N*. *meningitidis* (Fig 3).

**Fig 4:**
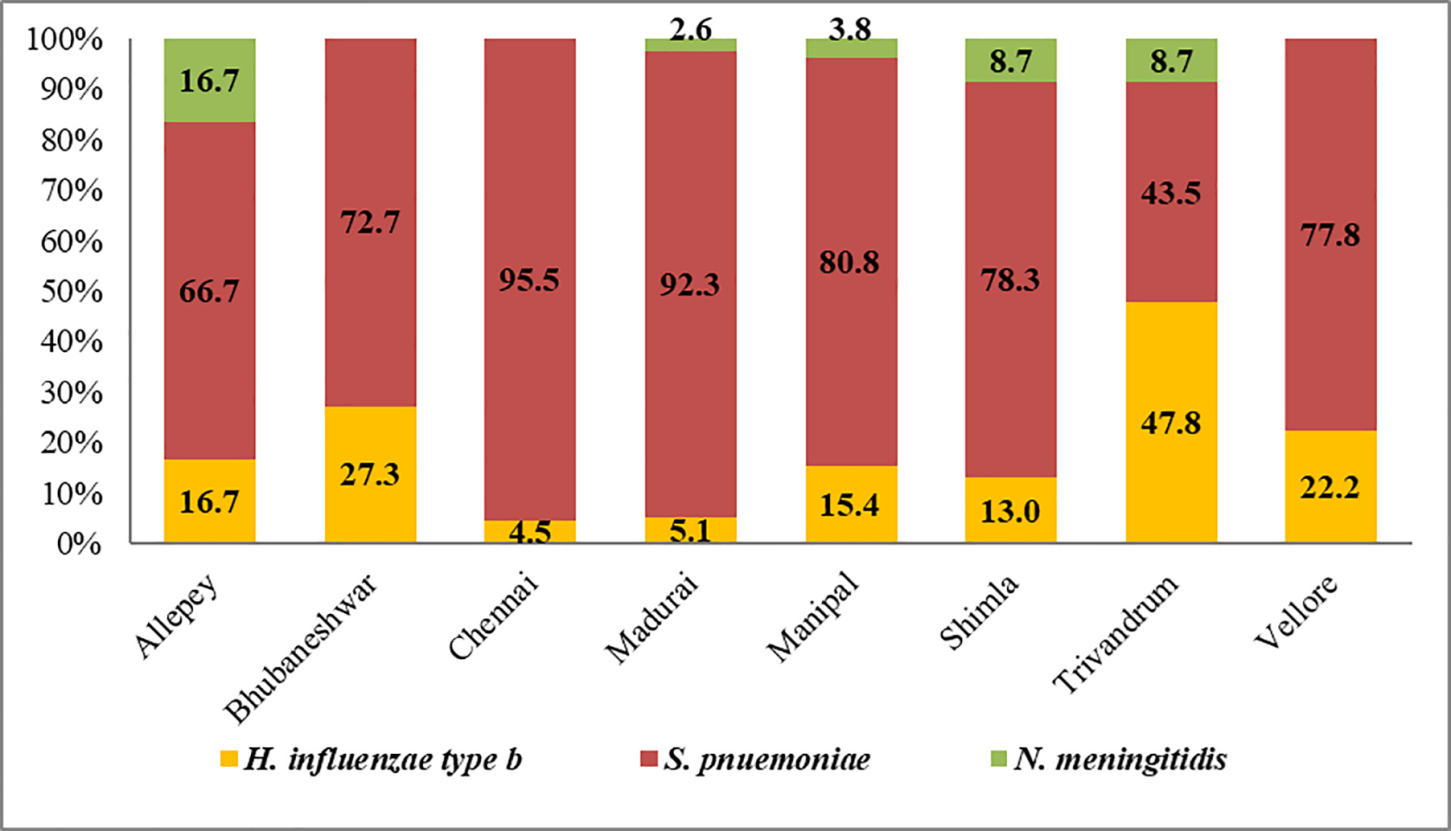
Distribution of bacterial meningitis cases in all HBSSBM sentinel sites.

Majority of the confirmed bacterial meningitis cases (77.4%; 199/257) occurred in children less than 2 years of age and 55.3% occurred in children aged between 1 and 11 months. Nearly 57.2% (147/257) of the confirmed cases were male children (Table 1). *S*. *pneumoniae* was the most frequent species identified in all sentinel sites except in Trivandrum, where *H*. *influenzae* type B (47.8%) was the predominant pathogen (Fig 4).

**Table 1:** Demographic profile of bacterial meningitis cases admitted in HBSSBM sentinel sites.

Variables	Level	Suspected cases (N = 3104)		Probable cases (N = 326)		Confirmed cases (N = 257)	
		n	%	n	%	n	%
**Age (months)**	1–11	1738	56.0	192	58.9	142	55.3
	12–33	722	23.3	51	15.6	57	22.2
	24–35	260	8.4	22	6.7	16	6.2
	36–47	196	6.3	22	6.7	19	7.4
	49–59	188	6.1	39	12.0	23	8.9
**Gender**	Male	1817	58.5	210	64.4	147	57.2
	Female	1287	41.5	116	35.6	110	42.8

**Fig 5:**
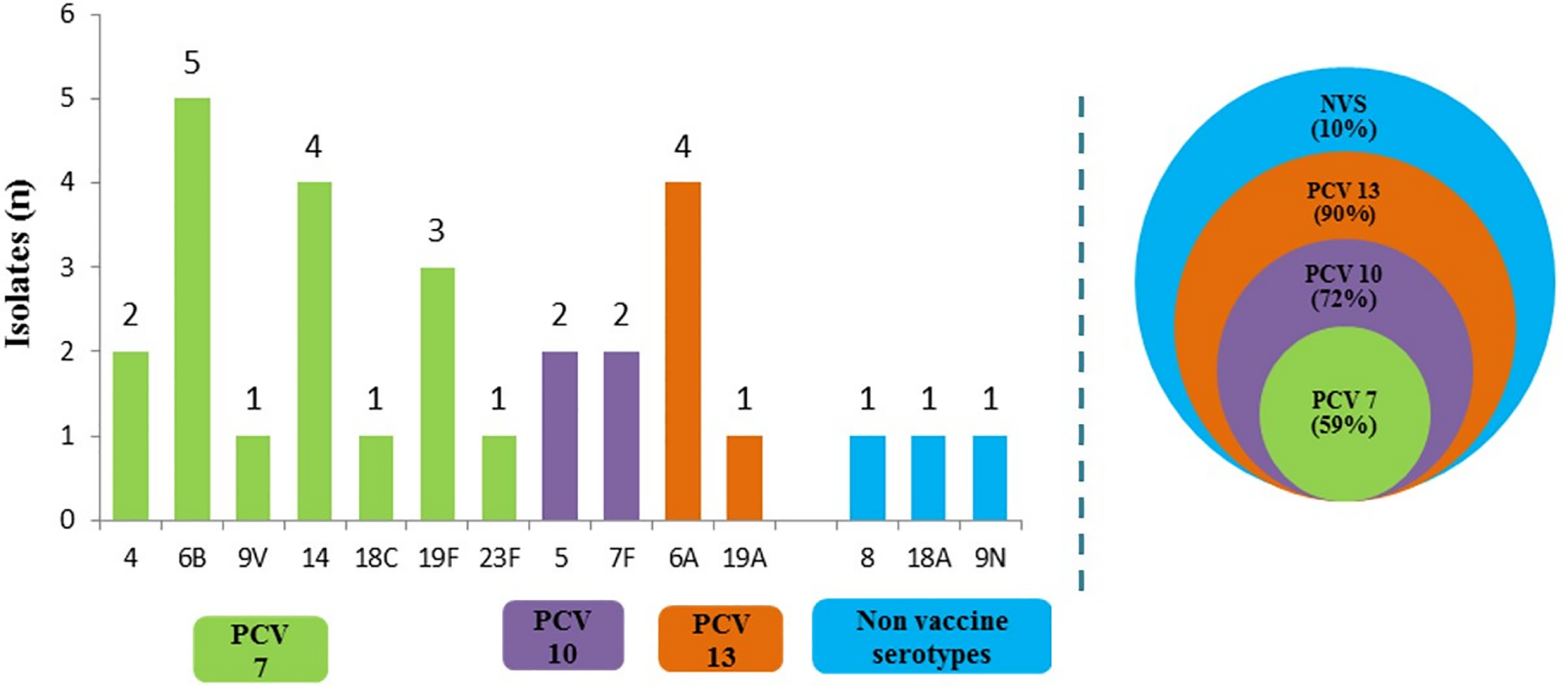
*Streptococcus pneumoniae* serotypes distribution between March 2012 and Feb 2013 (n = 29) and proportion covered by currently available PCV. PCV- Pneumococcal Conjugate Vaccine; NVS- Non Vaccine Serotypes.

The results of detection of *H*. *influenzae type b*, *S*. *pneumoniae* and *N*. *meningitidis* by various lab tests are summarized in Table 2. Among the cases of pneumococcal meningitis, *S*. *pneumoniae* was isolated in only 29 cases. Serotyping was performed on the 29 *S*. *pneumoniae* isolates and 14 different serotypes were identified (Fig 5). Ninety percent of pneumococcal meningitis cases were caused by serotypes covered by the PCV-13 in comparison to the serotype coverage by other PCV vaccines *viz*. PCV- 10 (72%) and PCV- 7 (59%). Ten percent of serotypes detected in the present analysis are not covered in any of the current PCVs.

**Table 2:** Laboratory confirmation of bacterial meningitis pathogens.

Test	*H*. *influenzae type b* (N = 37)		*S*. *pneumoniae* (N = 213)		*N*. *meningitidis* (N = 7)	
	n	%	n	%	n	%
**Latex Agglutination (LA)**	21	56.8	22	10.3	5	71.4
**Blood Culture**	2	5.4	19	8.9	0	0.0
**PCR Test**	1	2.7	155	72.8	0	0.0
**More than one test**						
**i. Culture + LA**	3	8.1	6	2.8	1	14.3
**ii. LA + PCR**	8	21.6	7	3.3	1	14.3
**iii. Culture + PCR**	1	2.7	2	0.9	0	0.0
**iv. Culture + LA + PCR**	1	2.7	2	0.9	0	0.0

**Fig 6:**
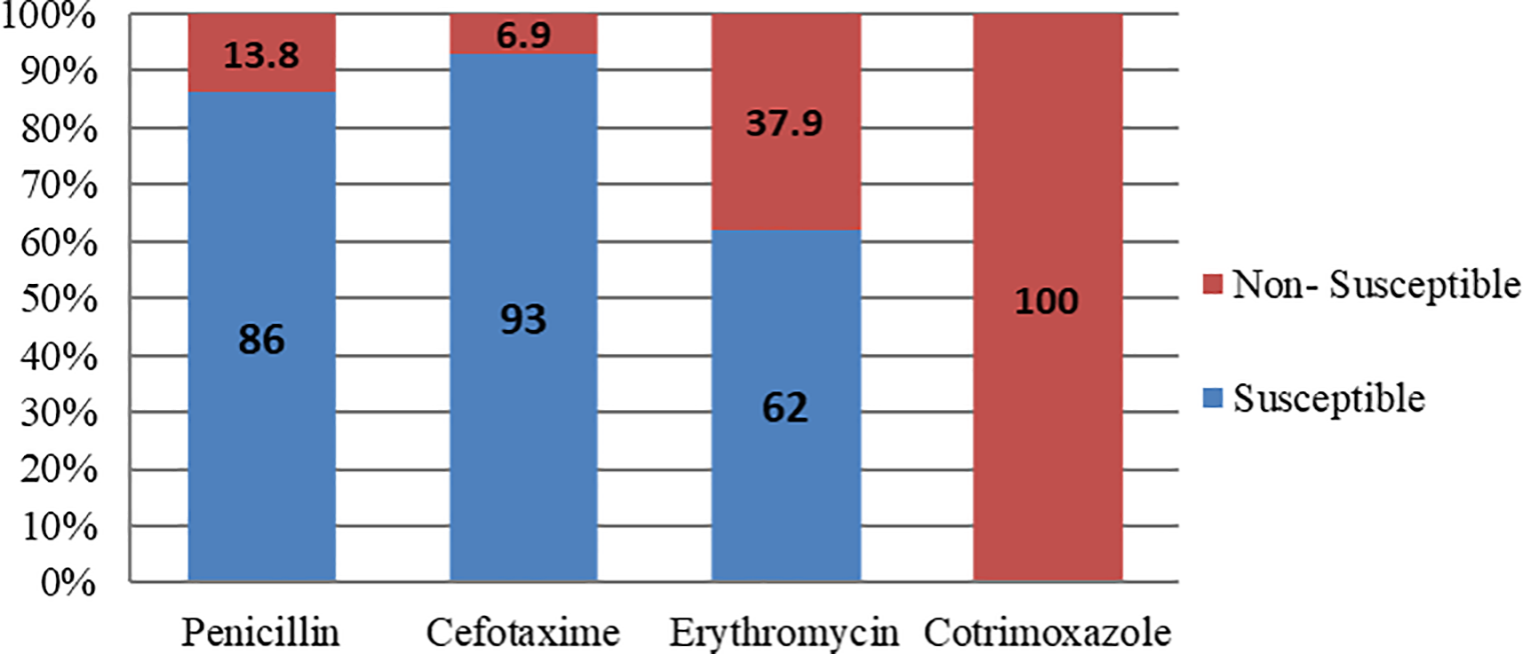
Antimicrobial susceptibility pattern of *Streptococcus pneumoniae* isolates (n = 29).

Antimicrobial susceptibility testing revealed that all 29 isolates were non-susceptible to cotrimoxazole and 11 isolates (37.9%) were non-susceptible to erythromycin. Four isolates (13.8%) were non-susceptible to penicillin, of which three isolates were non-susceptible to erythromycin. Two penicillin non-susceptible isolates were also non-susceptible to cefotaxime (Fig 6).

## Discussion

*H*. *influenzae* type b was the leading cause of bacterial meningitis with high mortality among confirmed cases, prior to the introduction of the pentavalent vaccine in India [12]. A multi-site hospital based sentinel surveillance for bacterial meningitis in children in the age group of 1 and 23 months conducted between 2008 and 2010, reported 70% positivity for *H*. *influenzae* type b amongst all CSF culture positive cases [13]. It was reported that 13% and 8% of the confirmed cases were due to *S*. *pneumoniae* and group B *Streptococcus* respectively. However, surveillance conducted between 2011 and 2015 documented the emergence of *S*. *pneumoniae* as the major cause of invasive bacterial diseases among children less than 5 years of age [8]. These findings are corroborated by the data from the present study wherein *S*. *pneumoniae* was the commonest etiology of the confirmed bacterial meningitis cases followed by *H*. *influenzae* type b. The only exception observed was at the Trivandrum site where *H*.*influenzae* type b was more frequently isolated.

The decrease in the incidence of *H*. *influenzae* type b with concomitant increase in pneumococcal infections can possibly reflect the impact of the addition of Hib vaccine as part of the Pentavalent vaccine in the UIP from 2012 in India. Immunization of young children might not only have conferred protection against invasive *H*. *influenzae* type b disease, but could also have indirectly provided protection to unvaccinated individuals through interruption of disease transmission thereby reducing risk of acquiring Hib infection [14, 15]. There is likelihood for a similar shift in levels of pneumococcal disease in the post-PCV introduction scenario. Meningococcal meningitis cases were also seen in the reporting period albeit at a low level (2.7%) when compared to the previous reports [15–17].

In the present study, maximum number of cases were seen in children aged between one and 11 months accounting for 55.3% of all positive cases. High rates of bacterial meningitis in children of the same age group have also been reported in other Indian studies [18, 19]. Under-developed immune system in these very young children could possibly be making them more susceptible to bacterial meningitis [18].

The male to female ratio among confirmed meningitis cases in the present study was 1.3:1 and the difference was not statistically significant (p = 0.625). Similar male preponderance has been reported in studies from Pune and Pondicherry wherein the male/female ratio was observed to be 1.8:1 and 1.4:1, respectively [19, 20]. It is a common societal practice in many communities in India that a male child gets preferential care [21]. Whether the hospitalization pattern is a reflection of this gender related differential access to health care even among very young children needs to be further explored.

The available evidence indicates low incidence of sporadic meningococcal disease in India when compared to *S*. *pneumoniae* and *H*. *influenzae* type b infections [22]. A recent review of published Indian studies has concluded that meningococcal disease in India is of low prevalence with occasional, but large, epidemics particularly in northern India [22]. In this report, meningococcal meningitis accounted for 2.7% of all the confirmed cases. An earlier study in six hospitals reported a low prevalence of meningococcal meningitis (1.5%) in India [23]. Thus, it is clearly evident that meningococcal disease needs to be monitored on robust surveillance platforms with wider geographic representation to more effectively map the burden of meningococcal infection in India.

Annually about 700,000 to 1 million deaths attributable to pneumococcal disease occur globally with majority of them occurring in the developing countries. Hence, in 2007 WHO recommended the inclusion of pneumococcal conjugate vaccine, PCV7 in the national immunization programs [24]. Although PCV7 covered 80 percent of pneumococcal serotypes causing invasive disease in the most developed countries, due to strain variation of pneumococcal serotypes in India, it is expected that PCV7 can offer only 50% protective efficacy compared to the developed countries [25]. Currently licensed PCV10 and PCV13 formulations are likely to offer coverage for more serotypes prevalent in India and have been recommended by Indian Academy of Pediatrics (IAP) since 2012 [26]. The Government of India has planned to include PCV 13 in the UIP in the near future.

The major pneumococcal serotypes identified in this study included 6B (17.2%); 14 and 6A (13.8% each) and 19F (10.3%); and together they constituted nearly 55% of the 29 pneumococcal isolates. This finding is in agreement with the profile of pneumococcal serotypes recently published from India [8].

The successful management of bacterial meningitis depends on identification of the causative agent and appropriate antibiotic use [12]. There are reports of increasing drug resistance in *S*. *pneumoniae* worldwide. Very high prevalence of penicillin resistant *S*. *pneumoniae* has been reported from several countries with resistance rates ranging from 53.4% to 73.4% [27]. In the present study, however, we found that only 14% of the isolates demonstrated resistance to penicillin, and this finding was consistent with earlier reports from India [8, 28–30]. Similar to previous reports from the subcontinent, high resistance to cotrimoxazole and growing resistance to erythromycin is a point of concern as also evidenced in this study [2, 28, 29, 31, 32]. It would be advisable to generate pneumococcal serotyping data on more number of isolates to make more meaningful recommendations to the program.

## Conclusions

From the public health perspective, estimation of burden of bacterial meningitis by etiology is essential especially in settings where specific vaccines are yet to be introduced in the national immunization programmes. The present study describes the burden of bacterial meningitis among under five children in India and highlights the predominance of *S*. *pneumoniae* as an etiological agent, followed by *H*. *influenzae* and *N*. *meningitidis*. The study emphasizes the need for continued monitoring to understand the emerging patterns of antibiotic resistance and distribution and variety of pneumococcal serotypes in India. The possibility of replacement by currently prevalent and relevant pneumococcal serotypes at the vaccine manufacturer’s level will have to be considered in the event of decision on PCV introduction in the immunization program in India.

## References

[1] McIntyre PB, O'Brien KL, Greenwood B, van de Beek D. Effect of vaccine on bacterial meningitis worldwide. Lancet 2012 11 10;380 (9854):1703–11. doi: 10.1016/S0140-6736(12)61187-8 23141619 10.1016/S0140-6736(12)61187-8

[2] Ramakrishnan M, Ulland AJ, Steinhardt LC, Moïsi JC, Were F, Levine OS. Sequelae due to bacterial meningitis among African children: a systematic literature review.BMC Med 2009 9 14; 7:47 doi: 10.1186/1741-7015-7-47 19751516 10.1186/1741-7015-7-47 PMC2759956

[3] Edmond K, Clark A, Korczak VS, Sanderson C, Griffiths UK, Rudan I. Global and regional risk of disabling sequelae from bacterial meningitis: a systematic review and meta- analysis. Lancet Infect Dis 2010 5;10(5):317–28. doi: 10.1016/S1473-3099(10)70048-7 20417414 10.1016/S1473-3099(10)70048-7

[4] Scarborough M, Thwaites GE. The diagnosis and management of acute bacterial meningitis in resource–poor settings. Lancet Neurol 2008; 7: 637–648. doi: 10.1016/S1474-4422(08)70139-X 18565457 10.1016/S1474-4422(08)70139-X

[5] Jaiswal N, Singh M, Thumburu KK, Bharti B, Agarwal A, Kumar A, et al Burden of invasive pneumococcal disease in children aged 1 month to 12 years living in South Asia: a systematic review. PLoS ONE 2014 5 5;9(5):e96282 doi: 10.1371/journal.pone.0096282 24798424 10.1371/journal.pone.0096282 PMC4010478

[6] Mathew JL, Patwari AK, Gupta P, Shah D, Gera T, Gogia S, et al Acute respiratory infection and pneumonia in India: A systematic review of literature for advocacy and action: UNICEF- PHFI series on newborn and child health, India. Indian Pediatr 2011; 48: 191–218. 21478555 10.1007/s13312-011-0051-8

[7] Government of India. Operational guidelines: Introduction of Hib as Pentavalent Vaccine in Universal Immunization Program in India Ministry of Health and Family Welfare, Government of India, New Delhi, 2013.

[8] Manoharan A, Manchanda V, Balasubramanian S, Lalwani S, Modak M, Bai S, et al Invasive pneumococcal disease in children aged younger than 5 years in India: a surveillance study. Lancet Infect Dis 2017 3;17(3):305–312. doi: 10.1016/S1473-3099(16)30466-2 27956163 10.1016/S1473-3099(16)30466-2

[9] Sachdeva A. Pneumococcal Conjugate Vaccine Introduction in India’s Universal Immunization Program. Indian Pediatr 2017; 54(6): 445–446. 28667711 10.1007/s13312-017-1044-z

[10] Laboratory Methods for the Diagnosis of Meningitis caused by *Neisseria meningitidis*, *Streptococcus pneumonia*e and *Haemophilus influenzae*, WHO manual, 2^nd^ ed Geneva: World Health Organization 2011.

[11] Clinical and Laboratory Standards Institute (CLSI). Performance standards for antimicrobial susceptibility testing; 23rd informational supplement. CLSI document M100-S23. Wayne, PA: CLSI; 2013.

[12] Invasive Bacterial Infection Surveillance (IBIS) group, International Clinical Epidemiology Network (INCLEN). Prospective multicentre hospital surveillance of *Streptococcus pneumoniae* disease in India. Lancet 1999;353: 1216–21. 10217081

[13] Ramachandran P, Fitzwater SP, Aneja S, Verghese VP, Kumar V, Nedunchelian K, et al Prospective multi-centre sentinel surveillance for *Haemophilus influenzae* type b & other bacterial meningitis in Indian children. Indian J Med Res 2013;137(4):712–720. 23703338 PMC3724251

[14] Madhi SA. Pneumococcal conjugate vaccine and changing epidemiology of childhood bacterial meningitis. J Pediatr (Rio J) 2015;91:108–10. 25475554 10.1016/j.jped.2014.11.001

[15] Stephens DS. Protecting the herd: the remarkable effectiveness of the bacterial meningitis polysaccharide-protein conjugate vaccines in altering transmission dynamics. Trans Am Clin Climatol Assoc 2011; 122: 115–23. 21686214 PMC3116338

[16] Chinchankar N, Mane M, Bhave S, Bapat S, Bavdekar A, Pandit A, et al Diagnosis and outcome of acute bacterial meningitis in early childhood. Indian Pediatr 2002;39: 914–21. 12428036

[17] Grimwood K, Anderson P, Anderson V, Tan L, Nolan T. Twelve year outcomes following bacterial meningitis: further evidence for persisting effects. Arch Dis Child 2000; 83: 111–116. doi: 10.1136/adc.83.2.111 10906014 10.1136/adc.83.2.111 PMC1718445

[18] Mani R, Pradhan S, Nagarathna S, Wasiulla R, Chandramuki A. Bacteriological Profile of community acquired bacterial meningitis: A ten-year retrospective study in a tertiary neurocarecentre in South India. Indian J Med Microbiol 2007; 25(2): 108–14. 17582179 10.4103/0255-0857.32715

[19] Debnath DJ, Wanjpe A, Kakrani V, Singru S. Epidemiological study of acute bacterial meningitis in admitted children below twelve years of age in a tertiary care teaching hospital in Pune, India. Med J DY Patil Univ, 2012; 5: 28–30

[20] Bhat BV, Verma IC, Puri RK, Srinivasan S, Nalini P. A Profile of pyogenic meningitis in children. J Indian Med Assoc 1991; 89: 224–227. 1748798

[21] Singh A. Gender based within- household inequality in childhood immunization in India. Changes over time and across regions PLoS ONE 2012; 7(4): e35045 doi: 10.1371/journal.pone.0035045 22509379 10.1371/journal.pone.0035045 PMC3324412

[22] Sinclair D, Preziosi MP, John TJ, Greenwood B. The Epidemiology of meningococcal disease in India. Trop Med Int Health 2010;15(12): 1421–1435. doi: 10.1111/j.1365-3156.2010.02660.x 21054695 10.1111/j.1365-3156.2010.02660.x

[23] Kabra SK, Kumar P, Verma IC, Mukherjee D, Chowdhary BH, Sengupta S, et al Bacterial meningitis in India:an IJP survey. Indian J Pediatr 1991;58: 505–511. 1800332 10.1007/BF02750932

[24] World Health Organization. Pneumococcal conjugate vaccine for childhood immunization- WHO position paper. Wkly Epidemiol Rec 2007; 82: 93–104. 17380597

[25] Bravo LC, Asian Strategic Alliance for Pneumococcal Disease Prevention (ASAP) Working group. Overview of the disease burden of invasive pneumococcal disease in Asia. Vaccine 2009; 27: 7282–91. doi: 10.1016/j.vaccine.2009.04.046 19393708 10.1016/j.vaccine.2009.04.046

[26] Indian Academy of Pediatrics Committee on Immunization (IAPCOI). Consensus recommendation on immunization and IAP immunization timetable 2012. Indian Pediatr 2012;49: 549–64. 22885436 10.1007/s13312-012-0116-3

[27] Wang H, Huebner R, Chen M, Klugman K. Antibiotic susceptibility patterns of Streptococcus pneumonia in China and Comparison of MICs by agar dilution and E-test methods. Antimicrob Agents Chemother 1998; 42(10): 2633–2636. 9756768 10.1128/aac.42.10.2633 PMC105910

[28] Veeraraghavan B and Kurien T. Penicillin resistant *Streptococcus pneumoniae* in India: effects of new clinical laboratory standards institute breakpoint and implications. Indian J Med Microbiol 2011; 29(3):317–8. doi: 10.4103/0255-0857.83925 21860122 10.4103/0255-0857.83925

[29] Balaji V, Jayaraman R, Verghese VP, Baliga PR, Kurien T. Pneumococcal serotypes associated with invasive disease in under five children in India & implication for vaccine policy. Indian J Med Res 2015; 142: 286–92. doi: 10.4103/0971-5916.166588 26458344 10.4103/0971-5916.166588 PMC4669863

[30] Molander V, Elisson C, Balaji V, Backhaus E, John J, Vargheese R, et al Invasive pneumococcal infection in Vellore in Indi: Clinical characteristics and distribution of serotypes. BMC infect Dis 2013; 13: 532 doi: 10.1186/1471-2334-13-532 24206667 10.1186/1471-2334-13-532 PMC3827497

[31] Arifeen SE, Saha SK, Rahman S, Rahman KM, Rahman SM, Bari S, et al Invasive pneumococcal disease among children in rural Bangladesh: results from a population-based surveillance.Clin Infect Dis 2009; 48 (Suppl 2): S103–13. 19191605 10.1086/596543

[32] Batuwanthudawe R, Karunarathne K, Dassanayake M, de Silva S, Lalitha MK, Thomas K, et al Surveillance of invasive pneumococcal disease in Colombo, Sri Lanka. Clin Infect Dis 2009; 48 (Suppl 2): S136–40. 19191609 10.1086/596492

